# Hypergravity-assisted chemical liquid deposition of nano-granular film on the inner surface of a quartz tube

**DOI:** 10.1098/rsos.180722

**Published:** 2018-09-26

**Authors:** Y. K. Shen, X. Q. He, X. Gu, Z. Liu, Z. H. He

**Affiliations:** 1 School of Physics, Sun Yat-Sen University, Guangzhou 510275, People's Republic of China; 2 School of Chemical Engineering, Sun Yat-Sen University, Guangzhou 510275, People's Republic of China; 3 School of Engineering, Sun Yat-Sen University, Guangzhou 510275, People's Republic of China; 4 State Key Laboratory of Optoelectronic Materials and Technologies, Sun Yat-Sen University, Guangzhou 510275, People's Republic of China; 5 School of Physics and Astronomy, Sun Yat-Sen University, Zhuhai 519082, People's Republic of China; 6 School of Materials and Energy, Guangdong University of Technology, Guangzhou 510006, People's Republic of China

**Keywords:** hypergravity assisted, silver, granular thin film, inner surface, conductive window

## Abstract

Transparent tubes with functions of heating and temperature measurement are badly required in the visualization investigation of two-phase flows and flow-boiling heat transfer. In order to prepare such a tube, we introduced a cost-effective and energy-efficient procedure of hypergravity-assisted chemical liquid deposition (HACLD) to produce transparent and conductive silver (Ag) films on the inner surfaces of quartz tubes, typically 50 mm in length and 8 mm in inner diameter with a set-up that was designed and built for this purpose. Precursors of organometallic Ag precursor solutions were prepared by dissolving silver citrate and 1,2-diaminopropane in 2-methoxyethanol with required concentration for the chemical liquid deposition process. Semitransparent and conductive Ag films formed inside the required quartz tubes under specific heating process in hypergravity. One of the films was about 47 nm in thickness, 23 Ω per square sheet resistance, and 30% optical transmittance. This attempt may pave a way for the understanding of the film forming mechanism in hypergravity, and the development of a film preparation technology of HACLD.

## 1. Introduction

To investigate the flow-pattern dependent heat transfer and pressure drop of the two-phase flow, one needs direct observations of the flow patterns of nucleation and boiling in real time [1–3]. Transparent pressure-resistant quartz tubes are generally used as the windows for visualization experiment, intentionally with heating elements and temperature sensors mount on the outer wall of the tubes [4]. However, the thermal resistance between the inner surface and outer wall of the quartz tube blocks the accurate measurement of the temperature on the inner surface (the interface between the fluid and the tube) and results in a non-negligible error of the heat transfer coefficient, because of the heat leak of the heat source outside the tube. Heater on the inner surface of the tube can heat the fluid and measure the temperature of the heating wall more directly. This helps to reduce the heat leak and improve the accuracy of the local measurement of the temperature, and thus provide reliable parameters for the analysis. Therefore, it is necessary to develop a visual experimental tube with an effective heating surface that contacts with the fluid and measures the surface's temperature as well.

At present, the technologies of coating an inner surface of the tube generally adopt physical vapour deposition (PVD) [5–7], chemical vapour deposition (CVD) [8–11], electroplating [12,13], electrical explosion [14,15] and dip-coating [16–18]. They are summarized in table 1 for the comparisons of processes and sizes of the films. However, conventional coating methods have difficulties in implementing and fabricating uniform films inside a small tube, especially those with large aspect ratios. Methods of vapour deposition [19] face the challenge of concentration gradient of vapour; while electroplating [20] faces the challenges of the uniformity requirements of electric field and that of concentration of the solution. For an instant process of electrical explosion, there is not enough time to develop a uniform film structure. Dip-coating, one of the chemical liquid deposition, on the other hand, faces the challenges of ultra-thin liquid film development on a curved surface and in gravity, and the film surface management especially during the evaporation of the solvent. Spin-coating and ink-jet printing methods were applied to prepare conductive silver films on the glass surface [21]. However, the prepared silver films could not be thin enough to favour light transmission. Rotating the tube around its axis is helpful to improve the liquid distribution on the inner surface of the tube. When the rotation speed is high enough to produce centrifugal force much higher than the gravity (i.e. hypergravity to the rotating tube) on the inner surface of the tube, more advantages should appear for forming a uniform liquid film: (i) spreading out the liquid by overcoming the liquid surface tension; (ii) promoting the escape of the evaporated vapour (possibly bubbles) from the film, and (iii) accelerating the deposition of the once-produced solid particles onto the inner surface of the tube in the heating process. Therefore, one could expect to obtain thin films with increased particle density and improved particle adhesion on the inner surface of the tube.

**Table 1.** A list of comparisons of tube inside film preparation with different processes/techniques.

methods	year, author and reference	tube size and material	film material and thickness
PVD	1992, J. A. Sheward [5]	Ø120 mm, steel	Cr-Nb, 7–17 µm
	2016, D. Kottfer [7]	Ø200–300 mm, steel	Ti, 1.3–3.9 µm
CVD	1986, H. Itoh [10]	Ø10 mm, steel	TiN, 12 µm
	2013, H. Kousaka [9]	Ø4.4 mm, steel	DLC, 529 nm
	2014, R. Hatada [11]	Ø25 mm, steel	Ag-DLC, 150 nm
	2017, Y. Xu [8]	Ø0.9 mm, glass and lucalox	DLC, --
electroplating	2014, L. D. Sun [13]	Ø9 mm, Ti	TiO_2_, 5–15 µm
	2017, C. J. Xiang [12]	Ø10 mm, Ti	TiO_2_, 0.1–1 µm
electrical explosion	2000, O. Demokan [15]	Ø13 mm, Ta	Al, 7.1–8.1 µm
dip-coating	2007, W. C. Gu [17]	Ø50 mm, steel	ceramic, 300 µm
	2018, S. Ayata [18]	Ø16 mm, Al-Cu-Mg	ZrO_2_-DLC, 182 nm

In this paper, we developed a method of hypergravity-assisted chemical liquid deposition (HACLD) to prepare the required transparent heating tubes and characterized the structures and properties of the thus prepared films.

## 2. Experimental

Organometallic Ag precursor solution was the key precursor to prepare the Ag films. Five organometallic Ag precursor solutions were synthesized by using a modified complex reaction process [22] (see table 2 for the nominal Ag mass fraction and labelling). In the process of the Ag precursor solution preparation, Ag citrate, 1,2-diaminopropane and 2-methoxyethanol were used as the precursor compound, organic complexing agent and protective solvent, respectively. Taking the silver precursor solution SC as an example, 0.0078 M Ag citrate (C_6_H_5_Ag_3_O_7_, 4.0 g) and 0.0234 M 1,2-diaminopropane (C_3_H_10_N_2_, 1.7 g) were dissolved in 0.2933 M 2-methoxyethanol (C_3_H_8_O_2_, 22.3 g), stirred at 5°C for 10 min to ensure complete complexing, and then stirred at room temperature for 1 h to ensure a full chemical reaction. The ratio of the Ag to amine in the precursor solution was 1 : 3. At last, the transparent organometallic Ag precursor solution was obtained (figure 2*a*).

**Table 2.** Sample labelling.

solution	SA	SB	SC	SD	SF
nominal Ag mass fraction in solution (wt%)	1.0	2.0	3.0	4.0	5.0
heating at nominal 290°C, 3000 r.p.m.	FA	FB	FC	FD	FE
heating at nominal 340°C, 3000 r.p.m.	FA′	FB′	FC′	FD′	FE′

**Figure 1.**
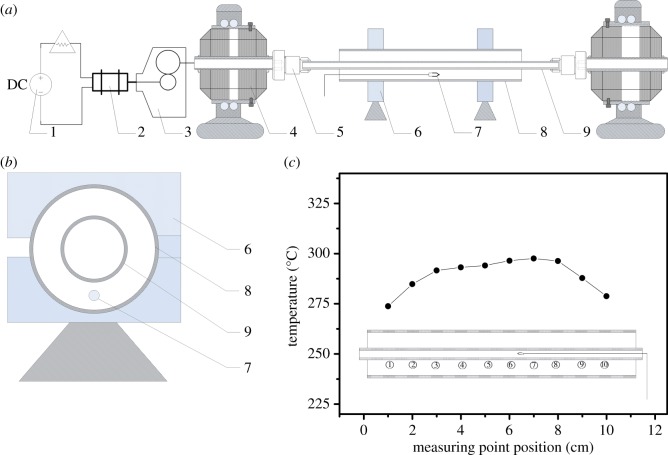
Schematic of the set-up for HACLD. (*a*,*b*) Drawing of the set-up. (1) DC power; (2) servo motor; (3) gearbox; (4) bearing; (5) tube clip; (6) ceramic base; (7) thermocouple; (8) transparent heating tube; (9) quartz tube. (*c*) Temperature distribution inside the quartz tube.

**Figure 2.**
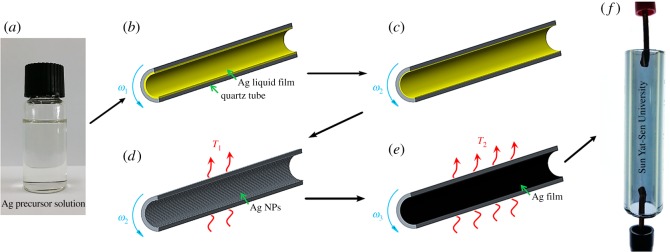
Schematics and process conditions of chemical liquid deposited uniform and transparent Ag film. (*a*) The synthesized organometallic Ag precursor solution; (*b*) Ag precursor solution spread out in the quartz tube; (*c*) Ag precursor solution liquid film in hypergravity; (*d*) Ag NPs formed at preheating temperature *T*_1_; (*e*) Ag film formed at heating temperature *T*_2_; (*f*) a photo of the film FC heated at 290°C.

A set-up was designed and built for the HACLD inside a tube (figure 1*a*). The quartz tube, with inner diameter of 8 mm and length of 200 mm, can be rotated by a DC servo motor at an adjustable speed (from 120 to 3000 r.p.m.) to provide different levels of gravity (from 1.6*g* to 40*g*, respectively) on the inner surface of the tube, and can be heated with a transparent heating tube (*D* = 23 mm, *L* = 110 mm) outside the rotating tube to allow in-site observation of the heat-treatment. The rotational speed of the quartz tube can be adjusted according to the colour of the film in the quartz tube which is observed through the transparent heating tube in real time. Hanging in the air, one end of a K-type thermocouple was installed between the quartz tube and the heating tube to indirectly measure the temperature of the rotating tube during the heat treatment. The temperature it took must be higher than that of the inner surface of the quartz tube (figure 1*b*). Calibration shows 7°C higher at the temperature of 290°C for static tube. The temperature distribution inside the heating tube with the static quartz tube shows that only the central part of 5 cm is uniform within 2% difference (figure 1*c*). Due to the limitation of the effective and uniform heating length of about 50 mm for the hypergravity set-up, all the results and discussions of the Ag films in this paper will be confined to this area. Figure 2 schematically illustrates the key procedure for the preparation of the uniform and transparent Ag films in the tube by HACLD.

**Figure 3.**
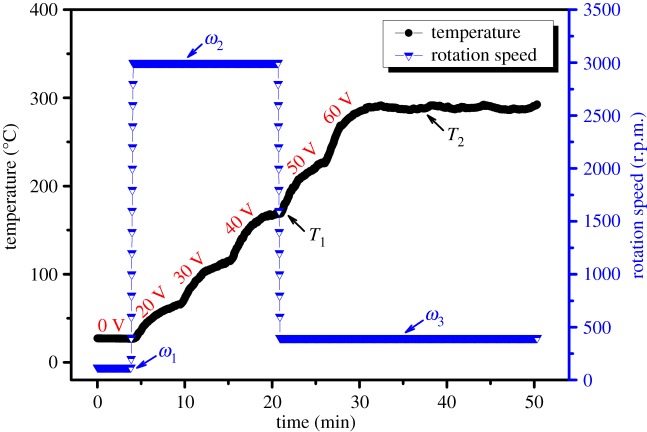
Profiles of rotation speed and nominal heat-treatment temperature for the preparation of the films.

The fabrication process consists of four phases (figure 2*b–e*). First, fix the quartz tube on the set-up, and inject the needed amount of Ag precursor solution into the tube; and then rotate the tube at the speed of 120 r.p.m. (*ω*_1_) for 5 min to ensure that the Ag precursor solution has spread out uniformly (figure 2*b*). Second, increase the rotation speed to 3000 r.p.m. (*ω*_2_) to create a hypergravity of 40*g* to make the liquid film even thinner (figure 2*c*). Third, preheat the quartz tube at the nominal temperature of 150°C (*T*_1_, taken by the K-type thermocouples) by using step-type AC voltage (20–40 V, shown in figure 3), which promotes the volatilization of the solvent and the decomposition of the solute in the liquid film (figure 2*d*). Assisted by the hypergravity, the Ag nanoparticles (NPs) once decomposed from the solution are accelerated to sink onto the inner surface of the quartz tube. Fourth, heat the quartz tube at the rotation speed to 400 r.p.m. (*ω*_3_) at the nominal temperature of 290°C or 340°C (*T*_2_) with step-type AC voltage (50–60 V, shown in figure 3) for 20 min (figure 2*e*). Ten films were prepared from the five Ag precursor solutions (table 2).

Differential scanning calorimetry–thermal gravimetric analyser (DSC-TGA) was used to study the thermal decomposition of the organometallic Ag precursor solutions, so as to determine the heating temperature on the film formation and determine the mass fraction of Ag in the solutions. The simultaneous thermal analyser (STA) 449F3 (Netzsch, Germany), with the mass measurement accuracy of ±1.5%, was employed for the DSC-TGA, with the heating rate of 10 K min^−1^ in flowing air. X-ray diffractometer D8 ADVANCE equipped with Cu K*α* radiation (Bruker, Germany), was used to perform X-ray diffraction (XRD), to characterize the crystal structures and the composition of the prepared films. For better comparison among different films, the fragments of cracked quartz tubes were pasted onto the test bench by using the Plasticine in the way that the quartz tube axis is parallel to the test bench and the bottom surface (on which X-ray sweeps) of the tube is at the same level of the surface of the bench with maximal error of ±0.2 mm. In this way, the error caused by the curvature of the inner surface of the quartz tube could be minimized to 0.31°. Scanning electron microscopy (SEM) images were performed on the field emission SEM Gemini500 (Zeiss/Bruker, Germany), to study the surface morphology of the prepared films. To characterize the relationship between the morphology and the optical and electrical properties of the films, a UV-3600 (Shimadzu, Japan) was used to measure the optical transmittances for nano-granular films on the pieces of broken tubes at room temperature; and a KDB-1 (KunDe, China) was used to measure the sheet resistance by a four-point probing system. At last, after a semi-transparent Ag film heater was prepared inside the quartz tube, a thermal camera A600 (FLIR Systems AB, Sweden) was used to take the infrared images of the surface of the tube heated by its conductive film. The voltage was applied by a DC power supply through copper wires at both ends of the film heater.

## 3. Results and discussion

**Figure 4.**
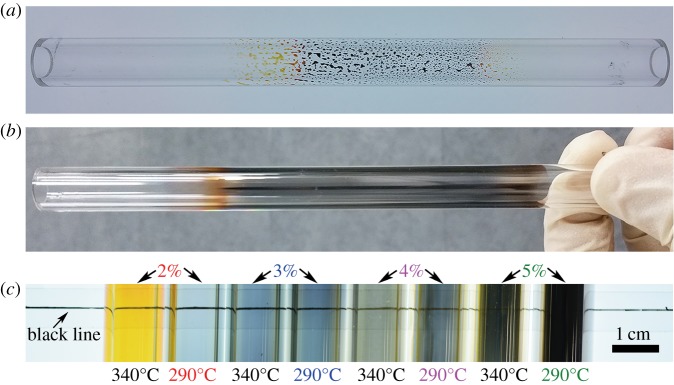
Digital photos of the as-prepared Ag film FC deposited on the inner surface of the quartz tube. (*a*) Heated at the rotation speed of 120 r.p.m. (*ω*_2_); (*b*) heated at the rotation speed of 3000 r.p.m. (*ω*_2_), the length of the prepared film in the middle of the tube is limited by the length of the heating tube; (*c*) Digital photos of the Ag films deposited on the inner surface of quartz tubes with different Ag precursor solutions, heated at 290°C and 340°C, respectively, and at the rotation speed of 3000 r.p.m.

Hypergravity was found essential to the preparation of thin films on the inner surface of the quartz tubes. In the attempt to obtain a suitable rotation speed for the film formation process, we heated the rotating tube with spread Ag precursor solution film at the nominal temperature of 290°C for 20 min and inspected the colour of the film simply with the naked eye. Take the solution SC as an example (see table 2 for the labelling), at low rotation speed of 120 r.p.m. during heating (*ω*_2_ in figure 3), the as-prepared sample looks like randomly distributed drops rather than film (figure 4*a*); while at 3000 r.p.m., the film was spread out (figure 4*b*), and formed uniformly and continuously as shown by the SEM image at the cross-section of the Ag film (FC in figure 7*c*). The digital photos in figure 4*c* show the films from SB to SE heated at 290°C and 340°C for 20 min, respectively. The black line drawn on the scotch tape behind the quartz tubes is for better comparison of the optical transmittance among the different quartz tubes. With the increase of Ag content in the precursor solution, the film colour changes from light blue to dark black, which may indicate the increases of the film thickness. The exception yellow colour of the film FB′ will be discussed later.

Correspondingly, with the increase of the Ag content in the precursor solution, the sheet resistance of the Ag film decreases dramatically (table 3). The decrease of the resistance could not be simply attributed to the increase of the thickness. The thickness of the film is 73 nm with sheet resistance of 2.1 Ω/□, while the thickness is 42 nm with 625.6 Ω/□.

**Table 3.** Geometric and physical properties of Ag films heated at nominal 290°C.

film label	FA	FB	FC	FD	FE
DGA mass fraction of Ag (wt%)	0.76	1.57	2.43	3.41	4.39
sheet resistance (Ω/□)	∞	625	23.5	4.1	2.1
film thickness (nm)	—	42	47	52	73
SEM surface coverage (%)	15	63	93	91	97
transmittance (%)	74	52	30	15	6

**Figure 5.**
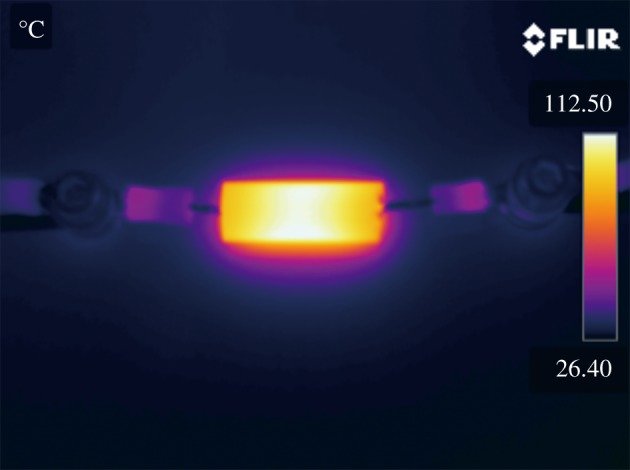
Infrared image of Ag FD heating tube showing the surface temperature distribution.

By applying a DC voltage of 6.0 V to the conductive film FC on the quartz tube, Ø8 mm × 50 mm in size, the surface temperature of the tube rises over 100°C, as shown by the infrared camera (FLIR, figure 5).

**Figure 6.**
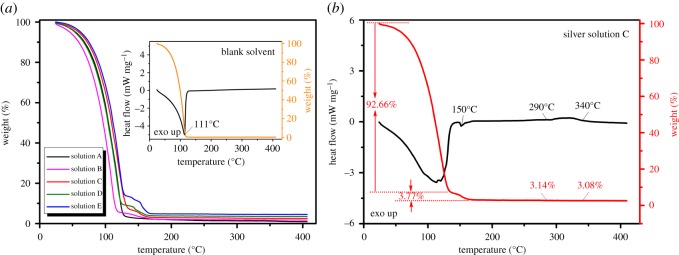
DSC-TGA curves to study the effect of heating temperature on the thermal decomposition of the organometallic Ag precursor solutions and the effect on the formation of the Ag films. (*a*) TGA curves for the organometallic Ag precursor solution (SA–SE). Inset: thermal behaviour of the reference sample (solvent only). (*b*) Thermal behaviour of solution SC (3 wt% nominal) in air at a heating rate of 10 K min^−1^.

Heating at suitable temperature can assist the evaporation of the solvent and promote the complete decomposition of the organic matter in the film. The TGA curves show that the five prepared organometallic Ag precursor solutions can completely evaporate at the temperature of 290°C (figure 6*a*). For comparison, the pure solvent completely evaporates at 111°C (as shown in the inset TGA curve of figure 6*a*). Take solution SC as an example, there are three stages of the reaction found in the DSC-TGA curves (figure 6*b*): (i) 70–120°C with a huge endothermic reaction accompanied with a large corresponding weight loss of about 93%; (ii) 140–160°C with an endothermic reaction appears and a small weight loss of about 4%, and (iii) 300–340°C with a gentle exothermic peak and almost negligible weight loss.

The first stage is mainly related to the evaporation of the solvent, which is similar to Nie *et al*.'s result [23]. The second stage must be crucial to the formation of the Ag films. It may be related to the silver precipitation from the solution to form Ag NPs. After subtracting the error (0.16%) caused by organic residues of the solvent evaporation (see the inset TGA curve of figure 6*a*), the final mass residue of Ag is 3.41% in this stage, more than the theoretical content (2.43%), which is probably due to the carbon retained. The formation reaction could be expressed by the following formula:

3.1 $$4{\left[\text{Ag}({\text{C}}_{3}{\text{H}}_{10}{\text{N}}_{2})\right]}_{3}{\text{C}}_{6}{\text{H}}_{5}{\text{O}}_{7}+\;15{\text{O}}_{2}\overset{\Delta }{⟶}12\text{Ag}↓+12{\text{C}}_{3}{\text{H}}_{10}{\text{N}}_{2}+24\text{C}{\text{O}}_{2}+10{\text{H}}_{2}\text{O}.$$

**Figure 7.**
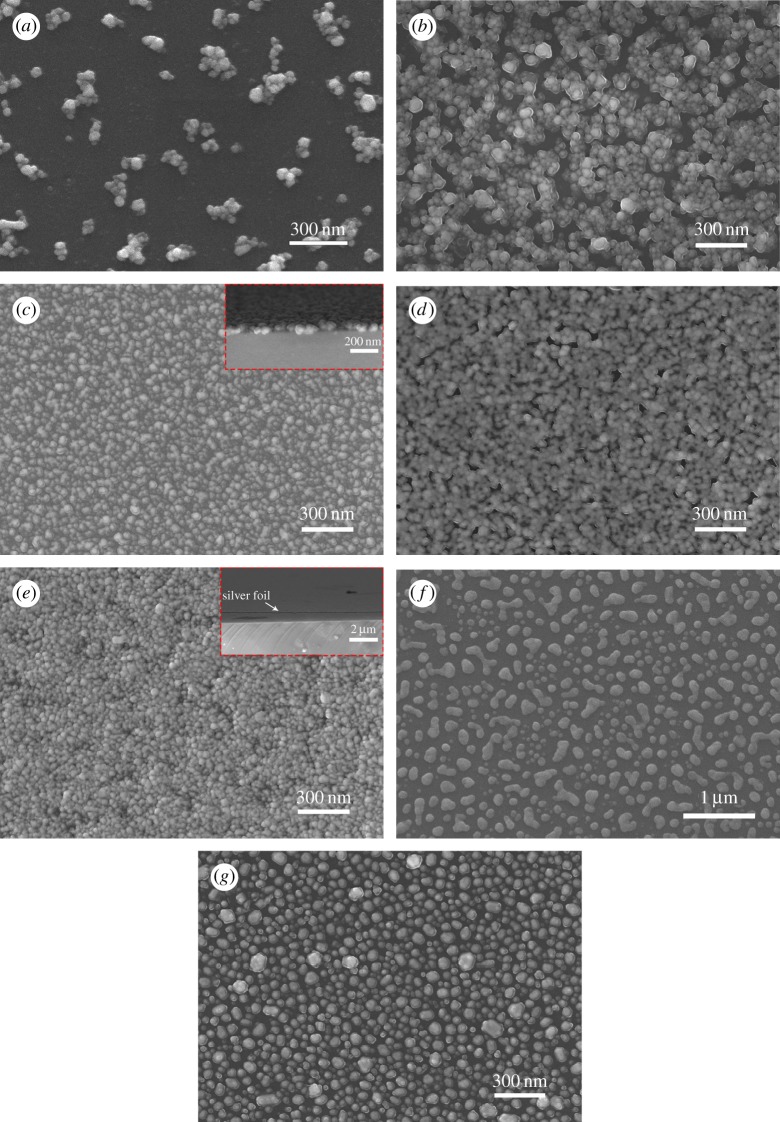
Electronic microscopy images of the Ag films. (*a*–*e*) SEM images of the Ag films (FA–FE) deposited on the inner surface of the quartz tube, heated at nominal 290°C. The inset SEM images in (*c*,*e*) show the cross-section of the Ag films. (*f*) SEM image of the Ag film FC′, heated at nominal 340°C. (*g*) SEM image of the Ag FB′, heated at nominal 340°C.

The temperature of the endothermic reaction (150°C) is much lower than the decomposition temperature of silver citrate powder (190°C), but agrees with the thermal decomposition behaviour of the silver citrate in solution shown by Nie *et al*. [23]. The gentle exothermic peak in the third stage (300–340°C) could be attributed to the fusion of the Ag NPs into much bigger drops. This is supported by the corresponding SEM images (shown in figure 7*c*,*f*), which show that the Ag film turns from the thin and dense granular structure at nominal 290°C, into a thick and drop-like structure at nominal 340°C.

SEM was performed to characterize the surface morphology at the stage of the growth of Ag films. The morphologies of films (FA to FE, FB′ and FC′) are shown by the SEM images in figure 7*a*–*g*. They are selected from 80 images of the representative samples. Isolated particle clusters are found for film FA, and evenly dispersed on the quartz substrate (figure 7*a*). For film FB, much more dense and better-connected particles are found with size of about 40–50 nm, comparable to those of film FA (figure 7*b*). Particles are fully connected with each other in film FC (figure 7*c*), with mean size that looks even smaller than those of FB. Particles seem to start to fuse into each other for film FD, supported by the merged boundaries and loose interspace in the image (figure 7*d*), as compared to film FC. The average particle size of film FD is comparable to that of film FC. Film FE shows the film thickness of about 70 nm with the apparent particle size of 40–50 nm (see figure 7*e* and its inset). Higher Ag content in the solution leads to the higher density of the Ag particles and larger thickness of the film, but has little effect on the size of the Ag particles (40–70 nm). All the films from FB to FE are of single-layer particles, as shown in the inset SEM cross-sectional image in figure 7*c*,*e*. By binarizing the SEM images with the threshold level of 110, the coverage of the silver particles in the film was obtained for each film and listed in table 3. By increasing the heating temperature to nominal 340°C, the particles turn into much bigger isolated drops (figure 7*f*).

**Figure 8.**
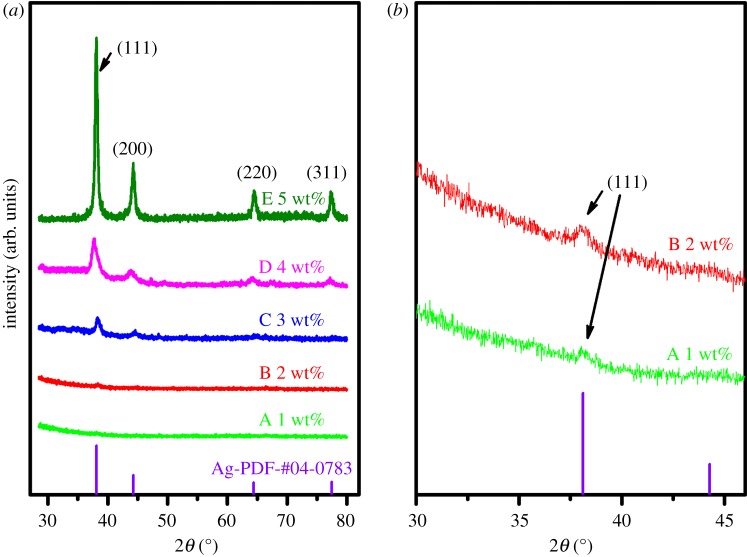
X-ray diffraction to study the Ag films with different mass fraction of Ag. (*a*) XRD patterns for Ag films (FA–FE); (*b*) magnification of the XRD intensity for Ag films (FA and FB) with 20 accumulative scans.

X-ray diffraction (XRD) was performed for the films to characterize their crystal structure. Figure 8*a* shows the XRD patterns of the Ag films, heated at nominal 290°C for 20 min. No obvious diffraction peak is observed for the film FA, and only a weak diffraction peak appears at the position of the main peak in the film FB. Twenty accumulation scans for both film FA and FB show only a weak diffraction peak of (111) plane, but no trace of any of the silver oxide (figure 8*b*). With the increase of Ag content (FC and FD), the diffraction peaks of Ag appear gradually, and the intensity increases. All of the diffraction peaks of film FE match those of Ag (111), (200), (220) and (311) crystal planes (Ag-PDF # 04-0783). There is no other diffraction peak in the patterns. The small offsets of the diffraction peaks (2*θ* = +0.25° for film FB, +0.15° for film FC, −0.29° for film FD, and +0.01° for film FE, respectively) in the XRD patterns (figure 8) could be attributed to the curvature of the inner surface of the quartz tube, leading to the X-ray scanned surface being different from that of the test bench surface. They were within the error range of 0.31°. However, the intensity of the diffraction peak is not proportional to the thickness of the film, nor to the Ag content in the precursor solution that forms the film.

One can conclude that all the particles in the films (FC to FE) are in the same crystal structure, and with the same lattice constant, but might not be fully crystallized or there might be mass loss as suggested by the SEM, especially for that with low Ag content. The full width of the diffraction peak at half-maximum (FWHM 0.756) is almost the same for all the three films (figure 8), also suggesting that the particles are comparable in size (58 nm). This is in agreement with the SEM observation (figure 7).

**Figure 9.**
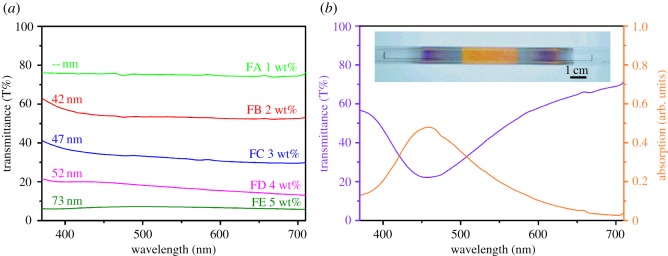
Transmittance spectra and absorption spectrum of the Ag films. (*a*) Changes in the UV/vis transmittance spectra of the five types of Ag film heated at nominal 290°C; (*b*) transmittance and absorption spectra of Ag FB′. Inset: digital photo of the Ag FB′ on the inner surface of the quartz tube, heated at nominal 340°C.

Optical transmittance of a single layer of the Ag film was measured to evaluate the application availability of the tubes. The transmittance decreases as the Ag content in the solution increases (figure 9*a*), and basically, is a little higher at shorter wavelength. This explains why the tubes with thin film are light blue in colour. The film FA shows the highest transmittance of 74%, and the film FE shows the lowest of 6%. The transmittance and the absorption spectra of film FB′ (figure 9*b*) tell why the tube is yellow in colour (see the photo in the inset). The length of the yellow region is about the same as that of the uniform area of the heating tube of the set-up (figure 1). The absorption spectrum is in close agreement with the absorption of the localized surface plasmon resonance (LSPR) (*λ*_SPR_ ≈ 458 nm) on the Ag NPs sizing 40 nm in the previous study [24]. This particle size is comparable to that of FB′ shown in figure 7*g*.

After analysing the films' properties, we may understand more on the role the hypergravity plays in assisting the formation of the nano-granular thin film. First, competitive with the surface tension, the hypergravity makes it possible to control the thickness of the liquid film of the Ag precursor solution, which determines the thickness of Ag film. Second, during the heat treatment process the surface tension becomes weaker at the beginning as the temperature increases, the hypergravity compresses the liquid film to become even thinner and more uniform, before the solvent evaporation that may increase the surface tension and viscosity of the liquid. Then, by offering much higher buoyancy, it is beneficial to the escapes of smaller bubbles of the volatilized solvent vapour and the gas produced by the decomposition of the organic matter (Ag citrate) in the liquid film, as those found by Zhang *et al.* [25]. Finally, it makes the once-formed Ag NPs (with much higher density than that of the liquid) in liquid film to sink more quickly to the bottom of the liquid film. This is also more conducive for the sinking particles to collect more Ag from the remaining organic compounds in liquid film, which assists faster growth of the NPs. In short, it helps to form the thin film and improve its surface roughness and uniformity, making possible a heating window of quartz tube.

HACLD provides alternative film preparation technology from that of spin coating. First, spin coating can only apply to a flat surface, and it requires weak surface tension solution, even though the uneven tangential force that applied along the substrate surface may tears the liquid film apart. HACLD, on the other hand, offers the centrifugal force (*ω*^2^*r*) perpendicular to the substrate surface; it fits the liquid film to the circular surface, thins the liquid film by pressure that competes with the surface tension, and prevents the liquid film from tearing apart. Then, heat treatment is only in normal gravity for the spin coating, but in hypergravity plus normal gravity for HACLD where the normal gravity becomes a periodical disturbance (*g*sin(*ωt*)) on the hypergravity. Taking into account the ineluctable vibration (noise) of the set-up, the force added on the film is given by:

3.2 $$G_{⊥}=\omega^{2}r+g\text{sin}(\omega t)+\overset{n}{\underset{k=0}{\sum }}a_{k⊥}\text{sin}(k\omega t)$$

and

3.3 $$G_{∥}=\overset{n}{\underset{k=0}{\sum }}a_{k∥}\text{sin}(k\omega t),$$

where *ω* is the angular frequency, *r* is the inner radius of the tube, *g* is the normal gravity acceleration, *t* is the time of process and *k* is an integer. In other words, the vibration disturbance always accompanies the hypergravity. It could help to organize the granular structure of the micro- and even the nanoparticles. More investigation is needed to understand the mechanism of the combined effects of periodical disturbance and hypergravity on the formation process of the nano-granular structure of the growing particles.

## 4. Conclusion

With the self-designed and home-made set-up, we developed a technique of hypergravity-assisted chemical liquid deposition for thin film preparation on the inner surface of quartz tubes. Semi-transparent and uniform silver films were thus obtained, with suitable electric resistance for the application of heating windows for experiments of fluid mechanics. Micro-structure characterization showed that the films were of nano-granular structure with particle size of 40–70 nm. More researches are expected to understand the combined effects of periodical disturbance and hypergravity, and even the chemical reaction on the motion of vapour bubbles, the growth of the particles, and the organization of the granular structure. Optimization among the film properties is needed for specific applications. It also paves a way to expand the technology of HACLD to the preparations of other films.

## Supplementary Material

Supplementary material.rar

## Data accessibility

The datasets supporting this article have been uploaded as part of the electronic supplementary material.
